# Subcutaneous Sacrococcygeal Myxopapillary Ependymoma in Asian Female:A Case Report

**DOI:** 10.4021/jocmr678w

**Published:** 2012-01-17

**Authors:** Kyung-Jae Lee, Byung-Woo Min, Hyuk-Jun Seo, Chul-Hyun Cho

**Affiliations:** a Department of Orthopaedic Surgery, College of Medicine, Keimyung University, Daegu, Korea

## Abstract

Subcutaneous sacrococcygeal myxopapillary ependymoma is extremely rare tumor that has a tendency to develop in children and adolescents. There have been several case reports and sporadic reports in the literature. However, no case has been reported in an Asian patient, to the best of our knowledge. We describe a 25-year-old Asian female patient with a subcutaneous sacrococcygeal myxopapillary ependymoma that had been clinically diagnosed as a pilonidal cyst. The tumor was treated successfully by surgical excision and the patient is doing well without evidence of local recurrence or distant metastasis at 2 years after surgery.

**Keywords:**

Myxopapillary ependymoma; Subcutaneous; Sacrococcygeal

## Introduction

Ependymomas are slowly growing glial cancers of the central nervous system that account for over 60% of spinal tumors of glial origin [1]. However, they rarely occur outside of the central nervous system known as extraspinal ependymomas. The majority of the extraspinal ependymomas occur in the sacrococcygeal subcutaneous tissue or the presacral regions [2-7]. Although the incidences of ependymoma do not differ with respect to race [8], no case of subcutaneous sacrococcygeal myxopapillary ependymoma has been reported in an Asian patient. We present a case of an ependymoma arising from the sacrococcygeal subcutaneous tissue in a 25-year-old Asian female.

## Case Report

A previously healthy 25-year-old-Asian woman was referred for evaluation of an enlarging, painless, subcutaneous mass located intergluteal fold. It was clinically diagnosed as a pilonidal cyst by previous examiner. She could remember that a mass had been present in this region for 2 years. During the 2-year period the mass had slowly increased in size from less than 0.5 cm to over 2.5 cm in diameter. During the past few months, the mass had become tender and sometimes making it uncomfortable to sit down. There was no history of urinary or faecal problems. Physical examination revealed a 2 × 3 cm sized, solid, mildly tender, mobile mass over the coccyx. It was well circumscribed proximally but not in distally on palpation. Neurological examination was normal.

**Figure 1:**
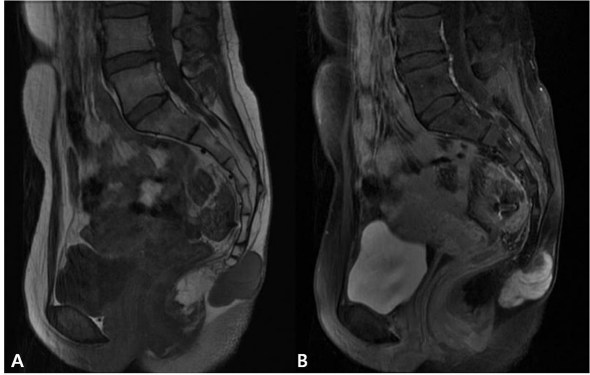
MR image shows the well circumscribed mass near the coccyx. The tumor was lobulated with low signal T1-weighted (A) and high signal T2-weighted image (B).

Magnetic resonance imaging (MRI) revealed a 6 ×3 cm well-circumscribed subcutaneous mass located over the coccyx. This tumor was comprised of two ovoid mass and there was no direct invasion to the coccyx (Fig. 1). Ultrasonography (USG)-guided needle biopsy was performed and microscopic examination showed a myxopapillary ependymoma. Computed tomography (CT) scanning of the thorax and abdomen demonstrated no evidence of lung or liver metastases. Isotope bone scanning revealed no evidence of skeletal metastases.

**Figure 2:**
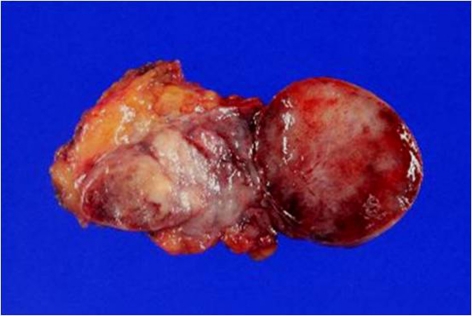
Photograph of sectioned gross specimen shows a dark red to brown soft tissue tumor which composed of two ovoid mass.

**Figure 3:**
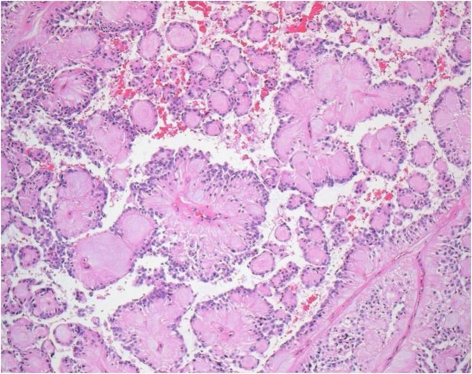
Photomicrograph demonstrates a columnar cells surrounding vascular cores. (H & E, × 200)

The mass was completely excised. Intraoperatively, the lesion was seen near the tip of the coccyx and well-circumscribed. It was composed of two ovoid but contiguous masses, the larger 3.0 × 2.5 × 1.5 cm, and the smaller 1.2 ×1.0 ×0.8 cm (Fig. 2). The histology of the specimens again confirmed a myxopapillary ependymoma (Fig. 3). Her postoperative course was uneventful and no adjuvant therapy was offered.

**Figure 4:**
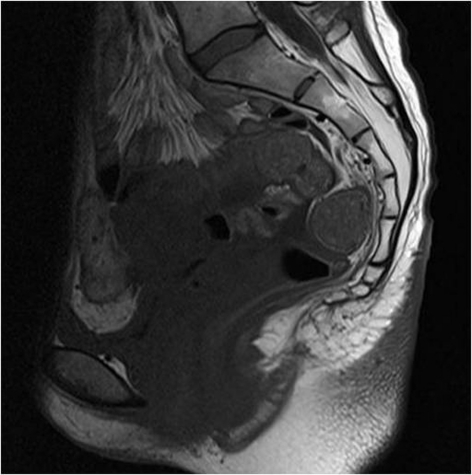
MR image taken 2 years after operation shows no local recurrence.

During 2 years of follow-up no local recurrence or metastasis were detected, with chest radiography and abdomino-pelvic MRI (Fig. 4).

## Discussion

Because ependymal cells may be found within the coccygeal ligament as well as in heterotopic position and thus ependymomas may rarely occur in extraspinal locations. Extraspinal ependymomas may be found in four general situations: metastatic extension from a primary central nervous system neoplasm; direct extension into the soft tissue of the sacrococcygeal area from primary ependymoma of the spinal cord, filium terminale, or cauda equina; primary tumor of the skin and subcutaneous tissue without any demonstrable connection with the spinal cord; and primary presacral, pelvic, or abdominal tumor [5,9]. Our case would correspond to the third situation of this classification.

Since the original description of an extraspinal ependymoma by Mallory in 1902 [4], there have been several case reports and sporadic reports in the literature [2, 3, 5-7]. With the review of literature, subcutaneous sacrococcygeal myxopapillary ependymomas are usually presented as a slow-growing mass in the intergluteal fold and often mistaken for a pilonidal cyst or other benign mass. The differential diagnosis includes pilonidal disease, teratoma, lipoma, chordoma, myxoid chondrocarcinoma, metastatic mucoid carcinoma and metastatic carcinoid [7]. Our patient also clinically diagnosed as a pilonical cyst.

The most important treatment for cure lies with complete surgical excision at the initial operation if possible. Coccygectomy and adjuvant radiotherapy may be needed if the tumor is attached to coccyx, in case of incomplete excision, or metastatic cases [2,7]. Long-term and close follow-up should be needed, because these tumors may recur locally or become metastatic. Sonneland et al. [9] reported that the extrameningeal ependymomas tend to metastasize more frequently than their intrameningeal counterparts which originate in the cauda. Also Helwig and Stern [5] reported four (17%) of 23 patients developed metastases and two of the metastasized patient died from progressive pulmonary and pleural metastasis. The best chance of survival of any patient with this type of tumor is achieved by complete excision at the time of the first operation [10].

We report a case of a subcutaneous sacrococcygeal myxopapillary ependymoma in an Asian female. To our knowledge, there has been no previous report of a case in an Asian patient. Although very rare, extraspinal myxopapillary ependymomas must be considered in the differential diagnosis of postsacral mass lesion. And postoperatively, long-term follow-up should be needed for evaluation of metastasis.
